# Medical Progress

**Published:** 1894-02-17

**Authors:** 


### Medical Progress.

### NERVOUS DISEASES.

*Epilepsy* (1).—It is doubtful whether the therapeutics of epilepsy are based even now on anything more than empiricism, so much divergence of opinion still exists as to the most effective treatment. Féré,[1] confirming his earlier statement that LARGE DOSES OF BROMIDES succeed when smaller ones have failed, records twenty cases treated for two or more years with daily doses of 4 to 5 drachms of an alkaline bromide or bromide of strontium; of this number only two did not improve. A patient taking such large doses needs to be carefully watched; skin affection, considerable loss of weight, mental depression, or subnormal temperature being developed, call for attention to the condition of the gastro-intestinal tract, as these symptoms of bromism may quickly assume a pernicious character, and their appearance demands the stoppage of the bromide, the excretion of the drug being hastened by purgatives. Eulenburg[2] prefers a mixture of the bromides of potassium, sodium, and ammonium, administered in dilute solution in water, saturated with carbonic acid, in doses of from 75 grains to 2½ drachms a day, in all cases shortly after a meal, and the treatment continued for two or three years after the occurrence of the last seizure. Much attention has been paid again of late to the value of BORAX in epilepsy, either alone or combined with bromides. Dr. Welch[3] records a case in which long continued bromide treatment had led to the depraved health of mind and body not unfrequently seen under this *régime*, and which under free diluents and 10-grain doses of borax thrice daily at first, and afterwards once daily, very greatly improved. It proved to be important to administer chemically pure borax, and not the impure commercial article. Dr. Welch, in reviewing the literature of the subject, remarks that borax is of most use in cases where the attacks are nocturnal in their occurrence, but should be tried in the cases of diurnal attacks where bromides have failed. Dr. G. M. Hammond, New York, gives doses larger than those just mentioned, but considers if 2 drachms per diem are ineffectual larger doses still will also be useless. [It is necessary not to forget in the continued administration of borax that it is a remedy not without its occasional drawbacks. Skin eruption, sores on the lips, a condition of the gums like that caused by lead, diarrhœa, and baldness, temporary or permanent, have all been recorded as following the prolonged use of the substance.—Ed.]

While urging the harm done by the continued administration of bromides, Dr. Inglis,[4] Detroit, thinks they should be given in all cases in full doses to begin with, and discontinued if no good effect is promptly shown. He considers that ANTIFEBRIN and its analogues form efficient substitutes for bromides; in moderate or small doses they can be given for long periods without deleterious effect upon the mental state.

Dr. Bates[5] draws attention once more to the powerful effect of NITRO-GLYCERINE in warding off, or rapidly terminating an epileptic paroxysm when administered hypodermatically in dose of 1-100 of a grain.

[We cannot but think that, given the morbid reflex excitability of the particular nerve centres, both the essential etiology of the epileptic attack, and the treatment of the epileptic condition in non-traumatic cases, are a question, first, of excess or diminution of arterial tension; second, of absorption in undue quantity, from the intestinal canal of bacterial toxines, skatol, and allied substances; and, third, the insufficient though copious elimination by the kidneys of ethereal sulphates, uric acid, and urinary toxines. Bromides simply diminish the irritability of the nerve centres; it is in cases of diminished arterial tension that Welch (*v. supra*) thinks that borax is successful, while antifebrin and its analogues can only act by maintaining a diminished arterial tension. Certainly we have seen remarkable recovery from prolonged hebetude, following the *status epilepticus*, where the pulse was slow and the arterial tension high, under the use of sodium nitrite; and, as regards the third point, it has been shown by experiments that the urine of an epileptic subject is abnormally toxic, while in our habitual attention to the diet and digestive apparatus of our epileptic patients, we empirically recognise the potency of toxines absorbed from the intestinal canal in inducing the convulsive attacks. The rational treatment of epilepsy might be to a large extent indicated by a physiologist and physiological chemist.—Ed.]

*Exophthalmic Goître* (1).—The view that this condition is dependent upon an excessive quantity of the internal secretion of the thyroid gland being thrown into the circulation continues to gain ground. Mr. Arthur Maude,[6] at the Medical Society of London, insisted upon the constancy in this disease of some degree of enlargement of the thyroid gland, in opposition to the idea that the disease is uncommon in goîtrous subjects. He considers that all the symptoms are consistent with the hypothesis of a general nerve poisoning, falling principally upon the medulla, and urged that the presence of goître, the relation to myxœdema, and the results of operations upon the thyroid gland justify the theory that the secretion of the thyroid gland is the origin of the mischief. Byrom Bramwell has called attention[7] to the exact contrast in the symptoms of myxœdema and exophthalmic goître, as also has Dr. G. R. Murray;[8] the latter comments upon the fact of myxœdema having replaced exophthalmic goître in some cases of the latter in which the thryroid gland has been removed by operation, and the bearing this fact has upon the etiology of Graves' disease.

On the other hand J. J. Putman,[9] in an article on the treatment of exophthalmic goître by thyroidectomy, withholds his assent to the doctrine of excessive internal secretion being the cause of the disease, and considers it to be a neurosis, the enlarged thyroid becoming a centre for the irritation of important nerves. Giving an analysis of fifty-one cases which have been operated upon, he still refrains from deciding which of the various operative procedures is the best; operation seems to cure or relieve about 90 per cent. of the cases subjected to that mode of treatment. It is possible, however, that surgeons in recording their cases have not always distinguished between the exophthalmic and other forms of goître.

Professor Greenfield,[10] in discussing Graves' disease as a disease of the thyroid gland rather than of the nervous system, is conscious that, with the exception of Byrom Bramwell, he is opposed in view to nearly all English and American physicians of eminence, although in accord with Möbius, Wette, Joffroy, Achard, and other authorities on the Continent. He arrays in support of this view the following considerations:—

1. Enlargement of the gland is due to a true but peculiar proliferation of gland tissue, unlike that seen in other goîtres, and as if designed for the performance of increased function.

2. The relief which is afforded by the removal, partial or entire, of the gland.

3. The presence in the nervous system of structural changes of a like character to those seen in toxic diseases, such as hydrophobia and tetanus, suggesting that they also may be of toxic origin.

4. The contrast in many of the leading conditions of myxœdema and Graves' disease, in respect both of anatomical changes as well as of symptoms.

5. The correspondence of the phenomena of Graves' disease with those produced by the artificial introduction of the thyroid secretion.

He details with much skill and elaboration the contrast both in the pathological anatomy in the nerve centres, as well as in physical and mental symptoms, between myxœdema and Graves' disease, records the considerable degenerative changes to be found, especially in the nerve trunks of the lower limbs, suggests that tachycardia is possibly not a nerve phenomenon at all, but due to direct irritation of the heart muscle by the thyroid secretion, points out that while the myxœdematous are extremely predisposed to tubercle, cases of Graves' disease are almost—if not entirely—exempt from it, and concludes that exophthalmic goître must, in consequence of these various considerations, now no longer be classed with diseases which are primarily of the nervous system.

*Friedreich's Disease* (1).—Senator[10] has recently expressed the opinion that this disease is primarily due to congenital atrophy of the cerebellum, leading to secondary degeneration in the spinal cord. Chauffard,[11] however, records a case which, besides the ordinary symptoms, was attended with athetoid attitudes on movement, but without the usual choreiform instability, and in which the gait was not at all cerebellar in type. The pathogenesis, and perhaps also the essential morbid anatomy, he considers as yet unknown. Dr. Alex. James[12] also records a case in which the gait was truly tabetic in character, except that as usual Romberg's symptom was absent. He calls attention to the point that if, as habitually stated, there is sclerosis of the postero-median columns, as well as of other tracts of the cord, how is it in this disease that the transmission of the impulses of tactile impression is not interfered with? Do, indeed, the postero-median columns transmit cutaneous and tactile sensibility? In neither Chauffard's nor Alex. James's case was there any history of hereditary transmission; both were sporadic cases.

*Locomotor Ataxy.*—Dr. W. R. Gowers[13] not unnecessarily reminds us that Todd recognised the disease fifteen years before Duchenne particularly described it. He points out that the ataxy is not dependent upon, or in any way proportional to the degree of anæsthesia of the skin that may be present, but depends probably upon interference in the transmission of those afferent impulses which in normal degree are not represented in consciousness, derived mainly from the muscles and to a less extent from the joints, and which pass up the posterior median columns to the posterior median nucleus of the medulla, whence again they probably are continued to the middle lobe of the cerebellum. The simulation of this disease by chronic multiple neuritis, by some forms of general paralysis of the insane (in which indeed this spinal disease is really present as well as the cerebral disease), by the ataxic form of diphtheritic palsy, by cerebellar tumour, and by the unsteadiness of gait seen in some hysterical girls is pointed out. *Inter alia* Dr. Gowers expresses his present belief that the knee-jerk is never absent in healthy persons, contrary to the opinion he has formerly held. In respect of treatment he assigns little value to iodide of potassium except when given early in cases of acute course. Mercury, except in small tonic doses, he thinks distinctly injurious. Arsenic he considers of most value, and chloride of aluminium in doses of from two to four grains three times a day. Above all things he lays stress upon the bladder being watched, to make sure that the viscus is completely emptied at least once in the twenty-four hours. This in the later stages of the disease frequently ceases to be the case without catheterism, and death in many, if not most examples of the malady is due to the results of putrefaction of urine within the bladder.

Dr. B. Sachs[14], in emphasising the fact of syphilis being the prevailing cause of locomotor ataxy, and of other conditions resembling that disease, calls attention—first, to the frequency with which syphilitic leptomeningitis is to be found post-mortem, as well as the posterior spinal sclerosis; second, that the change in the posterior root-fibres is most developed between the ganglia upon those roots and the cord itself—the place, that is, where the meninges come most closely into relation with the root-fibres; and, third, that antisyphilitic treatment is productive of amelioration in a considerable proportion of cases. Dinkler, reporting upon Erb's cases, gives good proof of improvement in 58 per cent. of cases of tabes.

In contrast to Erb, Leyden[15] some time ago endeavoured to show that antisyphilitic treatment was ineffectual in tabes, this view has not received general support. Werner Stark[16] records a case with syphilitic history in which, small doses of iodide having failed, the drug was administered in increasingly large doses until the quantity of twelve grammes per diem was reached; the treatment having lasted, with intervals of omission, for over two years, great improvement in the symptoms was observed.

Dr. H. F. Hansell[17] in an article on the ocular phenomena of early tabes, calls attention to the point, that (in addition to the well-known transient paralysis of the external muscles of the eyes, to the occasional myosis, to the Argyll-Robertson pupil, and of the peculiar mode of response of the myotic pupil to mydriatics) the first sign of altered function of the optic nerve is limitation of the colour field without narrowing of the field for white, deterioration of central acuteness of vision for form, or ophthalmoscopic changes. Later, the field for white becomes concentrically limited, central vision becomes less acute, and the opthalmoscope shows the well-known characteristics of non-inflammatory atrophy. While there is no doubt that incipient atrophy may in some cases be the forerunner of tabes, in most cases the perimeter is superior to the opthalmoscope in early diagnosis.

[1] Rev. de Méd., March, 1893. [2] Therap. Monat. quoted in Clin. Journ., May 17, 1893. [3] New York Med. Journ., Nov. 25, 1893. [4] Therap. Gaz., Aug., 1993. [5] New York Med. Journ., July 29, 1893. [6] Brit. Med. Journ., Oct. 21, 1893. [7] Edin. Med. Journ., May, 1893. [8] Lancet, Nov. 11, 1893. [9] Journ. Nerv. and Ment. Dis., Dec., 1893. [10] Bradshaw Lecture, 1893. [11] Berlin. Klin. Woch., 1893, p. 489. [12] The Med. Week, Sept. 8, 1893. [13] Edin. Med. Journ., Dec., 1893. [14] Clin. Journ., Oct. 4 and 11, 1893. [15] New York Med. Journ., Jan. 6, 1894. [16] Journ. de Méd. de Paris, Sep. 25, 1893. [17] Neurol. Centralblatt, June 15, 1893. [18] Journ. of Nerv. and Ment. Dis., No. 4., 1893.

